# Microbiome and pancreatic cancer: time to think about chemotherapy

**DOI:** 10.1080/19490976.2024.2374596

**Published:** 2024-07-18

**Authors:** Juliana de Castilhos, Katharina Tillmanns, Jana Blessing, Arnelyn Laraño, Vadim Borisov, Christoph K. Stein-Thoeringer

**Affiliations:** a Translational Microbiome Research, Internal Medicine I and M3 Research Center, University Hospital Tuebingen, Tübingen, Germany; b Cluster of Excellence “Controlling Microbes to Fight Infections” (CMFI), University of Tuebingen, Tübingen, Germany

**Keywords:** Pancreatic Ductal Adenocarcinoma (PDAC), microbiome, chemotherapy efficacy, microbial metabolism, tumor microenvironment

## Abstract

Pancreatic ductal adenocarcinoma (PDAC) is a highly aggressive cancer characterized by late diagnosis, rapid progression, and a high mortality rate. Its complex biology, characterized by a dense, stromal tumor environment with an immunosuppressive milieu, contributes to resistance against standard treatments like chemotherapy and radiation. This comprehensive review explores the dynamic role of the microbiome in modulating chemotherapy efficacy and outcomes in PDAC. It delves into the microbiome’s impact on drug metabolism and resistance, and the interaction between microbial elements, drugs, and human biology. We also highlight the significance of specific bacterial species and microbial enzymes in influencing drug action and the immune response in the tumor microenvironment. Cutting-edge methodologies, including artificial intelligence, low-biomass microbiome analysis and patient-derived organoid models, are discussed, offering insights into the nuanced interactions between microbes and cancer cells. The potential of microbiome-based interventions as adjuncts to conventional PDAC treatments are discussed, paving the way for personalized therapy approaches. This review synthesizes recent research to provide an in-depth understanding of how the microbiome affects chemotherapy efficacy. It focuses on elucidating key mechanisms and identifying existing knowledge gaps. Addressing these gaps is crucial for enhancing personalized medicine and refining cancer treatment strategies, ultimately improving patient outcomes.

## 1. Introduction

The human microbiome, an intricate and diverse ecosystem distributed throughout various body sites, plays a pivotal role in orchestrating essential functions that affect both health and disease states.^1–4^ In recent years, emerging evidence underscores the critical role of the microbiome in carcinogenesis, showing that it is not only implicated in tumor development and progression, but also serves as a potential biomarker for early detection or for progression and treatment resistance in several types of cancer.^5–7^ Additionally, mounting evidence suggests that the microbiome has been shown to modulate the efficacy and toxicity of cancer immuno- and chemotherapies.^8–13^ Being aware of the dynamic interplay between the human microbiome in different types of cancer, it becomes necessary to uncover the specific microbiome interactions and mechanisms to eventually improve treatment efficacy and reduce treatment toxicity.^14,15^

Pancreatic ductal adenocarcinoma (PDAC) is currently the third most common cause of cancer-related deaths, and the incidence of this disease has been steadily increasing by approximately 1% each year.^16^ Despite significant advances in the treatment of PDAC, like the poly-chemotherapeutic regimens of FOLFIRINOX (5FU, leucovorin, irinotecan, and oxaliplatin) and the combination of gemcitabine and a nanoparticle albumin-bound paclitaxel (nab-paclitaxel), which have been reported to significantly improve overall survival, patients still have a poor prognosis with 5-year survival rates of 10% in the metastatic setting.^17–19^ This can be attributed to various factors (late-stage detection, aggressive tumor growth, frequent metastasis, and primary resistance to standard of care).^20,21^ Also, most of the chemotherapy protocols are associated with considerable toxicity, thus often preventing their application in elderly patients and/or patients with poor performance status.^22^ This highlights the need to find early and efficient treatments and biomarkers to predict responses or toxicity to chemotherapy.

In recent years, the role of the gut microbiome in modulating chemotherapy efficacy has gained significant attention. The intestinal microbiome exerts systemic effects by producing metabolites, and thereby modulating the innate and adaptive immune system or metabolic functions of the host. These bacteria can impact the bioavailability and metabolism of chemotherapeutic agents, thus affecting their efficacy.^23,24^ Intracellular bacteria within tumor cells, on the other hand, can directly influence tumor biology by modulating cell signaling pathways, immune evasion, and drug resistance. These bacteria might have unique adaptations that allow them to survive within the tumor microenvironment (TME), potentially altering the tumor’s response to chemotherapy.^25,26^ Therefore, while intracellular bacteria may have direct effects on tumor cells, intestinal microbiome may modulate the overall treatment response through indirect systemic effects. Understanding the distinct roles of intratumoral and intestinal microbiome is crucial for developing comprehensive therapeutic strategies.

Despite numerous studies highlighting the microbiome’s potential to influence drug metabolism, immune responses, and the TME, there remains substantial controversy regarding the extent and consistency of these effects. Some studies provide compelling evidence that specific bacterial enzymes can activate or deactivate chemotherapeutic drugs, thereby impacting their efficacy and toxicity. Conversely, other research findings are inconclusive or contradictory, often due to variations in study design, patient populations, and microbiome analysis methods. Understanding these complex and multifaceted interactions is crucial for optimizing therapeutic outcomes in PDAC. A comprehensive and balanced examination of the current evidence is essential to elucidate the microbiome’s true role in chemotherapy efficacy and to identify pathways for future research and clinical application. By exploring latest research, this review aims to provide a comprehensive overview of the key mechanisms, through which the microbiome could affect chemotherapy efficacy, and it aims to highlight current knowledge gaps. While this review focuses on chemotherapy, we acknowledge that the microbiome’s interactions with immunotherapy in gastrointestinal (GI) cancers are also critical. Therefore, we refer readers to existing reviews on microbiome-immunotherapy literature in GI cancers for a broader understanding of this topic. In addition, the review will also discuss cutting-edge methodologies, including machine learning tools, low-biomass microbiome analysis, and experimental models such as patient-derived organoids, which are crucial for advancing cancer therapy and diagnosis by providing deeper insights into the intricate interactions between microbes and cancer cells. Closing the knowledge gaps to fully understand the microbial contributions to chemotherapy efficacy holds immense potential for advancing personalized medicine strategies and refining therapeutic approaches to optimize cancer treatments.^27^

## 2. Mechanistic overview: interplay between microbial metabolism and efficacy of chemotherapeutic agents

Certain bacterial species in the gut can activate or deactivate drugs, influencing their bioavailability and therapeutic levels in the body. For instance, some bacteria can convert pro-drugs into their active forms or, conversely, deactivate drugs, reducing their effectiveness against cancer cells.^28,29^ The gut microbiome is known to impact drug pharmacokinetics and pharmacodynamics, notably through processes like metabolism and elimination.^30^ In recent research on treatment protocols for PDAC, there is a significant focus on the interactions between the tumor, immune cells, and the effects of various therapies, including chemotherapy.^31,32^ Building on the understanding of the gut microbiome’s influence on drug efficacy, and its role in modulating the effectiveness of therapies, especially in PDAC, the next section delves into the specific mechanisms of microbial metabolism and enzymatic degradation of chemotherapeutic drugs which can contribute to chemotherapy resistance.

### 2.1. Microbial metabolism and enzymatic degradation: dual forces shaping chemotherapy resistance and drug breakdown

Pharmacokinetic research focuses on the microbiome’s role in xenobiotic metabolism, especially regarding therapeutic drugs. Four key factors were identified: (i) enzyme secretion altering drug molecular structure, (ii) production of metabolites affecting drug metabolism, (iii) altering levels and functions of enzymes in the liver and intestines, and (iv) impacting the expression of human metabolic genes.^33,34^ Orally administered drugs pass through the gastrointestinal tract, encountering barriers like physio-chemical properties, transporters, enzymes, and bacteria, before systemic absorption.^30^ Gut microbial metabolism adds another layer of complexity, affecting drug bioavailability and pharmacokinetics.^35^ After that, drugs are systemically absorbed from intestines, or are expelled. They also undergo biliary secretion and reabsorption, involving enterohepatic recycling, a process linked to individual microbiome composition and drug efficacy.^36^ The gut microbiome plays a crucial role in drug efficacy and safety through its enzymatic activities, which can modify the structure, bioactivity, and toxicity of medications.^37^ Xie et al. (2020) and Zhao et al. (2023) identified key enzymatic actions such as reduction, cleavage, hydrolysis, and oxidation.^37,38^ These enzymatic processes modify the biochemical composition of drugs, thereby impacting their bioavailability, efficacy, and toxicity (Table 1).

**Table 1.** Microbial metabolism of chemotherapy drugs in PDAC.

Drug	Chemical structure	Microbial Enzyme	Potential Metabolic Reactions	Metabolite	Activity	Model	Clinical Impact	Ref.
Gemcitabine	Nucleoside analog	Cytidine deaminase (CDD)	Deamination of gemcitabine to its inactive form	dFdU	Inactive	*In vitro*, *in vivo*	Reduced efficacy	^8,39–41^
Irinotecan	Lactone ring, carbamate group	*β*-glucuronidase	Reactivation of of SN-38G back to SN-38 due to de- conjugation	SN-38	Active (toxic)	*In vitro*, *in vivo*	Increase toxicity	^42–46^
5-Fluorouracil (5-FU)	Pyrimidine analog	Bacterial homolog to the eukaryotic enzyme DPD	Redox reaction	DHFU	Inactive	Hypothetical	Decreased toxicity	^47–49^
Oxaliplatin	-	-	-	-	-	Hypothetical	-	^50^
Paclitaxel	Diterpenoid with a complex structure including a taxane ring and an esterside chain	Esterases, bacterial homolog to cytochrome P450en- zymes	Hydrolysis of ester bonds, oxidation	-	Altered	Hypothetical	Altered bioavail- ability and toxicity	^51–55^

**Figure 1.**
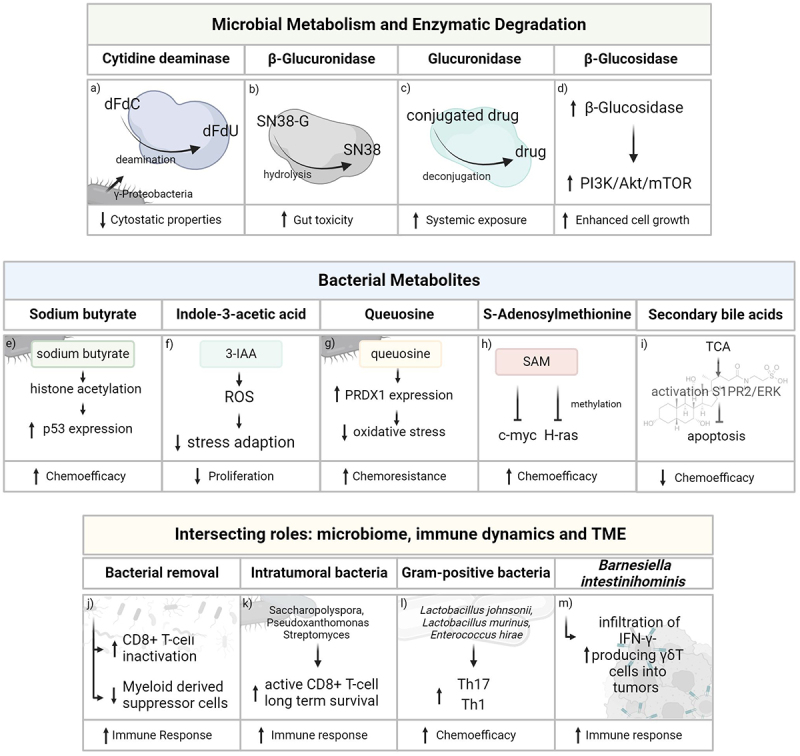
Microbial mechanisms in drug resistance. (a) The bacterial enzyme CDD deactivates active gemcitabine (dFdC) into inactive dFdU, reducing gemcitabine efficacy. (b) Bacterial *β*-glucuronidase reactivates irinotecans’ inactive prodrug SN38-G into toxic SN38, enhancing irinotecan-induced toxicity. (c) Glucuronidases are able to deconjugate glucuronidated drugs for elimination, enhancing their systemic exposure. (d) Enhanced expression of bacterial enzyme *β*-glucosidase in tumor tissue results in upregulated PI3K/Akt/mTOR signaling pathway activity, contributing to cancer progression. (e) Microbial butyrate can induce histone acetylation and expression of tumor-suppressor p53, leading to increased drug sensitivity in cancers lacking wild-type p53. (f) MPO oxidizes bacterial 3-IAA, which increases ROS in cancer cells, and reduces their stress adaption and proliferation ability. (g) The bacterial tRNA homologue queuosine can induce expression of PDX1, improving oxidative stress protection and thus reducing chemotherapy efficacy. (h) Bacterial SAM can reverse hypomethylation of the oncogenes c-myc and H-ras in cancer as well as enhance efficacy of chemotherapy in some cancers. (i) TCA can activate ERK signaling via the transmembrane receptor S1PR2, leading to regulation of expression of the apoptosis related proteins BCL2 and BAX, resulting in insensitivity of pancreatic cancer cells to gemcitabine. (j) Bacterial removal from cancer tissue increases CD8+ T cell activation and decreases amount of myeloid-derived suppressor cells. (k) Presence of specific intratumoral bacteria in long-term survivors is associated with increased T-cell activation. (l) Certain gram-positive commensals can boost number of T helper 17 and T helper 1 cells, thereby control cancer growth and increasing chemotherapy efficacy. (m) The gram-negative strain *Barnesiella intestinihominis* increases recruitment of IFN*γ*-producing *γδ*T cells into tumors enhancing anti-tumor immunity.

Abbreviations: CDD = cytidine deaminase; dfdU = 2’2’-difluoro-2‘-deoxyuridine; SN38-G = SN-38-Glucuronide; 3-IAA = 3-indoleacetic acid; SAM = Sadenoxylmethionine; ROS = reactive oxygen species; TCA = taurocholic acid.

As an example, Lehouritis et al. (2015) explored how gut microbes, such as *Escherichia coli* (*E. coli*), affect the efficacy of chemotherapy drugs like gemcitabine, which is frequently used in PDAC therapy.^39^ They showed that *E. coli* significantly reduces the effectiveness of gemcitabine and other chemotherapeutic agents *in vitro*. Specifically, *C*T26 tumor-bearing mice treated with gemcitabine plus *E. coli* displayed significantly increased tumor volumes compared to the gemcitabine only group at multiple time points. These findings suggest that the presence of certain bacteria in the gut and/or within tumors diminishes the anti-tumor efficacy of gemcitabine. This interaction between bacteria and gemcitabine is mediated by the bacterial enzyme cytidine deaminase (CDD), vital in nucleotide metabolism, especially in converting cytidine to uridine.^40^ This enzymatic activity significantly impacts essential cellular processes, including DNA and RNA synthesis. CDD deactivates the nucleoside analog gemcitabine by deamination, transforming it into an inactive form (2’,2’-difluoro-2’deoxyuridine (dFdU)) (Figure 1a), thereby substantially reducing the drug’s cytostatic properties. The role of bacteria in mediating gemcitabine resistance, especially through CDD, has been further investigated in studies by Voorde (2014)^41^ and Geller (2017).^8^

In PDAC, Geller et al. (2017) explored whether other bacterial species also confer resistance to gemcitabine.^8^ The authors hypothesized that intratumoral bacteria might be involved in the chemotherapy resistance mechanism because bacterial DNA was detected in 86/113 (76%) of PDAC samples and in only 3/20 (15%) normal pancreas controls. The majority of these bacteria were identified as Gammaproteobacteria, specifically from the Enterobacteriaceae and Pseudomonadaceae families. Subsequent analysis of 27 bacterial species revealed that 13 could induce resistance, with 12 species, primarily Gammaproteobacteria, possessing a long form of cytidine deaminase protein (CDDL) known to metabolize gemcitabine.

The gut microbial *β*-glucuronidase (gmGUS) is also a pivotal enzyme in the metabolism of various complex molecules, including hormones and drugs, as noted by Gao et al. (2022).^42^ This enzyme has been shown to be relevant for irinotecan induced toxicity as it reactivates the prodrug SN-38. Notably, the chemotherapeutic agent is a camptothecin derivative that contains a carbamate group that is deconjugated by gmGUS.^43^ The deconjugated prodrug then leads to severe intestinal side effects such as bleeding, diarrhea and acute weight loss^44–46,56^ (Figure 1b). Given irinotecan’s role in pancreatic cancer treatment, reducing these adverse effects is critical. Bhatt et al. (2020) conducted a study examining the consequences of inhibiting gmGUS activity. Targeted bacterial GUS inhibitors have been shown to partially alleviate irinotecan-induced GI tract damage and resultant diarrhea.^57^ Their findings indicated that targeted inhibitors of bacterial GUS could significantly mitigate gastrointestinal toxicity caused by enzymatically reactivated prodrug SN-38. This strategy not only reduced the gut damage induced by irinotecan, but also preserved the drug’s antitumor efficacy. This research presents a promising avenue for enhancing cancer treatment by modulating the activity of gut microbial enzymes.^45^

Beyond the deamination of gemcitabine by CDD and the deconjugation of the irinotecan prodrug SN-38 by gmGUS, knowledge regarding the metabolism of chemotherapeutics, particularly those used in PDAC therapy, remains limited. However, the potential for a drug to be metabolized by gut bacteria can be partially inferred from its chemical structure and the presence of functional groups commonly targeted by microbial enzymes. Important considerations include the specificity of these microbial enzymes and the prevalence of microbes that express them. For example, 5-Fluorouracil (5-FU), a pyrimidine analog, contains a fluorinated pyrimidine ring, which could potentially be targeted by microbial enzymes similar to those involved in nucleoside metabolism.^47^ Further, in humans, the protein dihydropyrimidine dehydrogenase (DPD) plays a critical role in drug metabolism, as it can detoxify 5-FU through metabolic conversion to dihydrofluorouracil (DHFU).^48^ The microbial strain *E. coli* was also shown to possess a functional homolog that can catalyze the conversion of uracil into 5,6-dihydrouracil *in vivo*.^49^ By identifying equivalents between known human and microbial mechanisms, potential metabolic reactions can be predicted in silico.

Another commonly used drug in the treatment of PDAC is oxaliplatin. This chemotherapeutic agent is a platinum-based compound with a 1,2-diaminocyclohexane (DACH) carrier ligand.^50^ To the best of our knowledge, there are no bacterial enzymes currently described that could alter the chemical structure of platinum-based drugs. This suggests that oxaliplatin is less likely to undergo significant microbial metabolism due to the absence of common microbial enzyme targets. Although direct metabolism by the microbiome might be limited, the gut microbiome can influence the efficacy and toxicity of oxaliplatin through indirect mechanisms. For example, the microbiome can modulate the host’s immune system, which in turn can affect the response to oxaliplatin.^2,58^ Certain gut microbes may enhance or diminish the drug’s effectiveness by modulating inflammatory responses or by influencing the integrity of the gut barrier, which can affect drug absorption and systemic availability.

Paclitaxel is utilized in the treatment of PDAC as part of a combination chemotherapy regimens (usually together with gemcitabine), to enhance therapeutic outcomes and improve patient survival.^59^ This chemotherapeutic agent is a diterpenoid pseudoalkaloid with a complex structure comprising a taxane ring and an ester side chain.^51^ The ester bond in paclitaxel is susceptible to hydrolysis by bacterial esterases, which may alter the drug’s activity.^52^ Additionally, paclitaxel is metabolized by cytochrome P450 enzymes in the liver.^53,54^ Given that cytochrome P450 enzymes are produced by both eukaryotes and prokaryotes, the presence of P450 enzyme-producing microbes could contribute to the metabolism of paclitaxel.^55^

Also, enterohepatic circulation, crucial in extending the pharmacological effects of certain drugs and their metabolites through biliary secretion and intestinal reabsorption, plays another significant role in drug bioavailability.^60^ Biliary secretion, primarily a drug elimination process, involves making drugs or their metabolites hydrophilic through glucuronidation, and reducing their reabsorbability.^30^ The gut microbiome, with its array of glucuronidase enzymes,^61^ can deconjugate these drugs, thereby modulating systemic exposure to both the drug and its metabolites^62^ (Figure 1c).

In addition to the direct interaction between chemotherapeutic agents and bacterial enzymes, which can alter chemotherapy efficacy, the microbiome can indirectly influence drug effectiveness by modulating immune responses and regulating eukaryotic signaling pathways. To broaden the scope of strategies for preventing chemoresistance, it is essential to focus on understanding these intricate microbe-host interactions. For example, *β*-glucosidase, an enzyme integral to numerous biological processes, particularly in the breakdown of complex carbohydrates, is produced by various bacteria, including lactic acid-producing bacteria in the human gut. The enzyme’s activities are crucial in different biochemical pathways, as outlined by Michlmayr and Kneifel (2014).^63^ In cancer, *β*-glucosidase can play a role in tumor growth.^64^ While *β*-glucosidase itself is not directly linked to chemotherapeutic drug metabolism in PDAC, its role in the broader context of glycoside metabolism can indirectly affect the efficacy of treatments, especially those involving glycoside-based prodrugs. The expression and activity of the enzyme *β*-glucosidase are notably increased in breast cancer tissues and cell lines as identified by Zhou et al. (2017).^64^ This upregulation activates the PI3K/Akt/mTOR signaling pathway, which contributes to enhanced cell growth (Figure 1d). Inhibition of the *β*-glucosidase was shown to significantly sensitize breast cancer cells to chemotherapy both *in vitro* and *in vivo*^64^, and might alleviate chemoresistance to 5-FU in gastric cancer.^65^ This research underscores a dual role and importance of *β*-glucosidase in cancer progression and its treatment.

In summary, the intricate interplay between microbial metabolism and enzymatic degradation significantly impacts the efficacy and resistance of chemotherapeutic agents. Our review underscores the necessity of considering the microbiome’s role in drug metabolism, highlighting the dual forces of bacterial enzyme activities and host-microbiome interactions. While several mechanisms of bacterial metabolism of chemotherapeutics have been uncovered, their applicability to PDAC remains is still unknown. Unraveling these mechanisms in PDAC could offer valuable insights for refining chemotherapy strategies and enhancing patient prognosis.

### 2.2. Bacterial metabolites: impact on chemotherapy efficacy and tumor progression

The fundamental role of microbial metabolites, and its complex assembly called the metabolome, in shaping physiological and pathological host functions, has already been recognized for many years, but only recently gained increased attention with advances of new “omics” technologies.^66,67^ These developments have uncovered the vast diversity of the metabolome, both between different individuals and within the same individual over time. This diversity is particularly important in a clinical context, as it might affect chemotherapy metabolisms and toxicity.^68^ Understanding these complex interactions between microbial metabolites and host functions is crucial for advancing personalized medicine and improving drug efficacy and safety.

Gut microbiota-derived metabolites are crucial in connecting the gut microbiome to cancer progression, modulating the effectiveness of chemotherapy drugs either by working synergistically to enhance the therapeutic effects or by inducing resistance. They can remodel the TME and regulate key signaling pathways in both cancer cells and immune cells.^69^ Within this environment, immune and inflammatory responses are predominantly driven by immune cells and cytokines, and microbial metabolites play a crucial role in modulating these responses which we will discuss in the following paragraph^70^:

Postler et al. (2017) classified microbial metabolites into three types: (1) Those derived directly from dietary components, like indole derivatives and short chain fatty acids (SCFAs); (2) host metabolites modified by gut microbiota, such as secondary bile acids; and (3) metabolites synthesized by microbes, for example, polysaccharide A.^71^ SCFAs, particularly butyrate, play vital roles in host physiology as signaling molecules, regulators of cellular metabolism, and modulators of immune responses.^1,72^ They can enter cells directly via diffusion or carrier-mediated transport.^73^ Additionally, some SCFAs functions involve binding to G protein-coupled receptors (GPCRs), including free fatty acid receptor 2 (FFAR2), FFAR3, and G-protein-coupled receptor 109a (GPR109a). Butyrate can act synergistically with chemotherapy drugs by inhibiting histone deacetylases (HDACs). This inhibition leads to increased histone acetylation, which affects the expression of genes involved in cell cycle regulation and apoptosis. Butyrate, in particular, has been shown to sensitize cancer cells to chemotherapy-induced apoptosis by modulating the expression of pro-apoptotic and anti-apoptotic proteins. This modulation enhances the therapeutic effects of drugs like irinotecan, doxorubicin, and adriamycin in colorectal cancer (CRC), multiple myeloma, and uterine cancers, respectively.^74–80^ Additionally, in gastric cancer, butyrate enhances the effects of cisplatin by increasing reactive oxygen species (ROS) within cells, reducing mitochondrial membrane potential (MMP), and inhibiting cell invasion and migration capabilities.^74,81^ On the other hand, butyrate has the ability to directly interfere with oncogenic signaling pathways, e.g., the JAK2/STAT3, vascular endothelial growth factor (VEGF), and Wnt pathways.^82,83^ By downregulating VEGF expression, for example, butyrate reduces angiogenesis, a critical process for tumor growth and metastasis. In addition, butyrate promotes the differentiation of cancer cells through the protein kinase C (PKC) pathway, making them more susceptible to chemotherapy.^82–84^ The combined effects of butyrate on histone modification, signaling pathways, and cellular differentiation make it multifaceted adjuvant in chemotherapy regimens.

Various studies have also investigated the impact of butyrate on pancreatic cancer cells. Its effects range from anti-proliferative effects, as highlighted by Bloom et al. (1989), Gaschott et al. (2001), Natoni et al. (2005), and Sanaei et al. (2022),^85–88^ to anti-invasive properties noted by Farrow et al. (2003).^89^ Additionally, its capability to enhance the efficacy of anticancer drugs has been demonstrated, as detailed in studies by Kitazono et al. (2010)^90^ and Panebianco et al. (2022).^91^ A critical aspect of butyrate’s action, particularly relevant in the context of chemotherapy resistance, is its interaction with the p53 protein. Pellegata (1994) suggested that the absence of functional p53 protein contributes to chemotherapy resistance.^92^ Kitazono et al. (2010) found that it can induce histone acetylation and the expression of the tumor-suppressor p53 in human pancreatic carcinoma cell lines lacking functional p53^93^ (Figure 1e). Moreover, they observed that combining butyrate with anticancer drugs like cisplatin and fluorouracil enhanced their effects. This discovery suggests new therapeutic strategies, especially for tumors with compromised p53 function, potentially addressing a key challenge in chemotherapy resistance. Panebianco et al. (2022) further explored the synergistic effects of butyrate and gemcitabine.^91^ Their research indicated enhanced drug efficacy in pancreatic cancer cell lines (human PxPC-3 and PANC-1) and xenograft mouse models when using a combination of these compounds, compared to gemcitabine alone. They observed that this combination not only inhibited cell growth but also intensified gemcitabine-induced apoptosis in cell cultures. Additionally, in mice, the combined treatment influenced the gut microbiome diversity, structure, and function, along with changes in the metabolome and lipidome. These changes included reduced levels of fatty acid amides and hydroxylated fatty acid metabolites, potentially impacting cancer metabolism and progression.

The microbial metabolite class of urolithins have also been linked to chemotherapy efficacy. They are derivatives of dibenzopyran-6-one produced gut microbes after consuming ellagitannin (ET)-rich foods like nuts, pomegranates, and berries. Microbes hydrolyzes ET to form ellagic acid (EA), which is then converted into urolithin A (UA) and urolithin B (UB) through processes of lactone-ring cleavage, decarboxylation, and dihydroxylation.^94–96^ Urolithin A improves the efficacy of 5-FU by modulating drug resistance pathways and reducing the expression of multidrug resistance proteins. Urolithin A (UA) can modulate of the forkhead box O3-forkhead box M1 axis, which leads to decreased expression of multi-drug resistance proteins (MRP) 2 and MRP7 on the cancer cell surface, thereby reducing the efflux of chemotherapy drugs in CRC, for instance.^97^ Similarly, UA downregulates breast cancer resistance protein, allowing mitoxantrone to remain in cancer cells longer, increasing its efficacy.^98^ On the other hand, urolithin B increases the toxicity of cisplatin and paclitaxel in esophageal cancer, although the exact mechanism remains unclear.^94^ These examples illustrate the potential of gut microbiome-derived metabolites to synergistically enhance or decrease chemotherapy efficacy through various mechanisms, including immune and epigenetic regulation.

In patients with PDAC, significant alterations in the microbiome and bacterial metabolome are observed with distinct variations between long- and short-term survivors. Riquelme et al. (2019) and Kiss et al. (2020) identified differing microbial profiles in these two groups.^99,100^ Building on this, Tintelnot et al. (2023) explored the relationship between treatment response and the gut microbiome in PDAC patients.^101^ They discovered a marked difference in the concentration of 3-indoleacetic acid (3-IAA) between patients responding and not responding to the FOLFIRINOX regimen. Higher levels of 3-IAA were found in patients and mice that showed a better response to this treatment. 3-IAA is synthesized from tryptophan by the intestinal microbiome using the enzyme tryptophanase.^102,103^ Tintelnot et al. demonstrated that 3-IAA enhances the efficacy of chemotherapy in PDAC.^101^ The key mechanism involves the enzyme myeloperoxidase (MPO), predominantly found in neutrophils, which oxidizes 3-IAA to produce ROS. The high ROS levels, induced by the MPO-mediated oxidation of 3-IAA, impair cancer cell stress adaptation mechanisms, particularly autophagy, leading to reduced cell proliferation and increased apoptosis (Figure 1f). In humanized gnotobiotic mouse models of PDAC, administration of 3-IAA along with chemotherapy significantly reduced tumor weight and improved treatment outcomes. This was further demonstrated by decreased tumor growth in mice colonized with responder microbiomes when treated with chemotherapy and 3-IAA.^101^ The study underscores the potential of microbiome-derived metabolites to augment the effectiveness of existing cancer therapies. While 3-IAA shows promising synergy with chemotherapy, there are caveats as indoles or other tryptophan metabolites can impair anti-tumor immune responses through the aryl hydrocarbon receptor (AhR) in PDAC.^101,104^ High AhR expression in human PDAC samples is associated with rapid disease progression and an immune-suppressive tumor microenvironment.^105^ Also, the beneficial effects of 3-IAA are highly dependent on the presence of MPO in neutrophils and neutrophil infiltration in tumors.^101,106^ This indicates that the effectiveness of 3-IAA could vary based on the patient’s immune cell profile and the specific tumor microenvironment.

In their research on the association between poor survival, therapy resistance in obese pancreatic cancer patients, and microbial metabolites, Kesh et al. (2022) focused on two microbial metabolites: Queuosine and S-adenosylmethionine (SAM).^107^ Queuosine, a bacterial t-RNA homolog, has been noted for its role in inhibiting cell proliferation and regulating metabolism.^108^ It is also linked to the induction of PRDX1, an antioxidant protein that protects tumor cells from chemotherapy induced oxidative stress^107^ (Figure 1g). Conversely, SAM is recognized for its anti-tumor properties, such as reversing the hypomethylation of oncogenes like c-myc and H-ras in gastric and colon cancers,^109^ and enhancing the efficacy of doxorubicin in breast cancer cells^110^ (Figure 1h). Kesh et al. analyzed the gut microbiome in obese mice resistant to gemcitabine and paclitaxel.^107^ They found a significant presence of bacteria producing queuosine in resistant mice with obesity, while lean, nonresistant mice had an enrichment of bacteria producing SAM. Supplementing obese animals with SAM sensitized pancreatic tumors to chemotherapy, and treating pancreatic cancer cells with queuosine increased PRDX1 expression. Additionally, tumors in obese mice had an increase in CD133+ treatment refractory pancreatic tumor populations compared to controls. The study revealed that queuosine accumulation in obese mice could protect tumors from chemotherapy-induced oxidative stress by upregulating PRDX1.^107^

The metabolism and toxicity of drugs can be significantly influenced by the competition between microbial metabolites and host proteins and enzymes, as explored in studies by Clayton et al. (2009) and Kaddurah-Daouk et al. (2011).^111,112^ Secondary bile acids, which are products of bacterial bile metabolism and modification, play a critical role in various diseases, including *Clostridioides difficile* infection, inflammatory bowel disease, metabolic diseases,^113^ and cancer.^114^ These bile acids are key in determining drug absorption from the intestine due to their involvement in drug solubilization, maintenance of supersaturation, modulation of lipase activity, and effects on the partitioning of ionized drugs. Additionally, they influence membrane permeability by affecting transporters and tight junctions.^115^ Malhotra et al. (2023) highlighted the vital interplay between bile acids and the gut microbiome in pancreatic cancer patients, who often show dysregulated bile acid levels and increased unconjugated bile acids.^116^ The influence of secondary bile acids on chemotherapy response is shown in detail by Yang et al. (2021).^117^ Increased expression of ABCA8, an ATP-binding cassette (ABC) transporter, on pancreatic cancer cells has previously been connected to poor prognosis in pancreatic cancer patients.^118^ In their paper, Yang et al. demonstrate the role of the secondary bile acid taurocholic acid (TCA) in TCA-S1PR2-ERK signaling-mediated apoptotic resistance, which plays a role in ABCA8-induced gemcitabine ineffectiveness^117^ (Figure 1i).

In summary, bacterial metabolites are pivotal in shaping the effectiveness of chemotherapy and influencing tumor growth. Recent advances in ”omics” technologies have uncovered the vast diversity of the metabolome, shedding light on how these metabolites affect drug metabolism, efficacy, and toxicity. Recognizing the interactions between microbial metabolites and host functions is crucial for pushing the boundaries of personalized medicine, with the potential to significantly improve therapeutic outcomes and patient prognoses.

### 2.3. Intersections of the microbiome, immune cell dynamics, and tumor microenvironment in chemotherapy response

The efficacy and toxicity of chemotherapy are not only influenced by bacterial enzymes and metabolites, but also by immune regulations. The gut microbiome plays a crucial role in modulating the host’s immune response and is integral to the development and education of the immune system.^2^ This modulation includes shaping the abundance, differentiation and activity of various immune cell populations, such as T cells, B cells, and antigen-presenting cells.^119–121^ For example, regulatory T cells (Tregs) are crucial in maintaining immune homeostasis but can also create an immunosuppressive TME that “protects” cancer cells. Tregs suppress anti-tumor immune responses by producing inhibitory cytokines like TGF-*β*, IL-10, and IL-35.^122^ Certain bacterial species can induce the development of Tregs, which further enhances this immunosuppressive environment, e.g., by suppressing effector T cell activity, and helps the tumor evade immune surveillance.^123^ Clinically, the presence of Tregs in the TME is associated with poor prognosis and reduced effectiveness of chemotherapy.^122,124^

Although many organs exhibit a diverse immune environment,^2^ PDAC presents a unique tumor entity as it is an immunologically “cold” tumor, possessing an immunosuppressive microenvironment. This characteristic, detailed by Gautam et al. (2023), contributes significantly to the poor response to therapies observed in PDAC.^125^ Pushalkar et al. (2018) linked these differences to immune suppression and carcinogenesis demonstrating that bacterial removal from cancerous pancreas tissue leads to immunological reprogramming involving a decrease in myeloid-derived suppressor cells and an increase in CD8+ T cell activation^126^ (Figure 1j). Further research by Riquelme et al. (2019) indicated that the tumor microbiome in long-term survivors of pancreatic cancer aids T-cell activation.^99^ This is associated with the presence of specific bacterial genera such as Saccharopolyspora, Pseudoxanthomonas, and Streptomyces within the tumor (Figure 1k). These findings suggest that the tumor microbiome composition is crucial in the anti-tumor immune response and could serve as a therapeutic target to enhance tumor sensitivity to treatments. In chemotherapy, some evidence emphasized the immunomodulatory effects of certain bacterial strains on treatment efficacy. Viaud et al. (2013) noted a modulation of immune cell populations and activation of pro-inflammatory genes vital for immune system stimulation after 48 hours following therapy with non-myeloablative doses of cyclophosphamide or the anthracycline doxorubicin in naive mice.^9^ This research underscores the importance of microbiome immunomodulation in cyclophosphamide treatments for PDAC. Cyclophosphamide is known not only for its direct anticancer properties, but also for its ability to stimulate anti-tumor immune responses, which can significantly enhance therapeutic efficacy.^127,128^ Viaud et al. (2013) specifically identified the role of certain gram-positive commensals, including *Lactobacillus johnsonii*, *Lactobacillus murinus*, and *Enterococcus hirae*, in boosting T helper 17 (Th17) and T helper 1 (Th1) cells, crucial in cancer growth control^9^ (Figure 1l). In studies involving MCA205 sarcoma-bearing mice, the use of vancomycin, which targets gram-positive bacteria, resulted in a reduced Th17 cell response and diminished the anti-tumor efficacy of cyclophosphamide.^9^ Further expanding on this, Daillere et al. (2016) discovered that cyclophosphamide’s immunomodulatory effects against cancer also involve the gram-negative strain *Barnesiella intestinihominis*. This bacteria aids in the recruitment of IFN-*γ*-producing *γδ* T cells into tumors^10^ (Figure 1m). These findings collectively indicate that both gram-positive and gram-negative bacteria within the gut microbiome are key players in modulating the immune response during cyclophosphamide treatment for cancer.

A diverse and balanced microbiome is associated with better health outcomes and survival.^99^ However, chemotherapy can also lead to dysbiosis, a microbial imbalance in the gastrointestinal tract, impacting immune response and chemotherapy effectiveness. Research, including studies by Lin et al. (2012), Montassier et al. (2015), Levy et al. (2017), Deleemans et al. (2019), Galloway-Pena et al. (2020), Rajagopala et al. (2020) and Wei et al. (2021), demonstrates that chemotherapy reduces microbiome diversity in feces of both animals and humans.^129–135^ Specific changes in bacterial species have been noted post-chemotherapy, such as the decrease in Firmicutes and Actinobacteria and increase in Proteobacteria observed by Montassier et al. (2015) in non-Hodgkin’s lymphoma patients.^130^ They also reported genus-level alterations, with bacteria linked to reduced inflammation decreasing and those associated with colitis increasing.

Although several studies highlight a significant shift in the abundance of individual bacterial species induced by chemotherapy, the specific effects of gemcitabine or FOLFIRINOX (two of the most used chemotherapy regimens in PDAC) on the gut microbiome during pancreatic cancer treatment are not fully understood. The impact of chemotherapy on the gut microbiome can be different between individuals, and influenced by factors such as the type of chemotherapy, treatment duration, and the individual’s baseline microbiome.^136,137^ Therefore, these microbial changes induced by chemotherapy impact: 1) the efficacy of chemotherapy by displacing beneficial bacterial species; 2) the side effects of treatment, such as chemotherapy-induced intestinal mucositis, by altering intestinal barrier function; and 3) immune modulation and function, potentially leading to dysregulation and an increased risk of infections.^130,131,138^ Disruption of this balance can result in immune dysregulation, thereby increasing the risk of infections diminishing the immune system’s efficacy against cancer cells and other threats.^135^

The gut microbiome is not only key to regulating immune responses, but also affects the success and side effects of chemotherapy drugs. Despite significant advances already made in this area, the interactions between the microbiome, immune system, and chemotherapeutic agents are highly complex and not fully understood. For example, the interactions between oxaliplatin, the epithelium, and the immune response in chemotherapy are highly complex and multifaceted. Oxaliplatin induces immunogenic cell death (ICD) in intestinal epithelial cells (IECs), particularly affecting the ileal crypts, which is critical for activating the immune response against colon cancer.^58^ The apoptosis of IECs triggers the release of damage-associated molecular patterns (DAMPs) that recruit and activate dendritic cells (DCs), which in turn present antigens to T cells and promote the accumulation of tumor-infiltrating lymphocytes (TILs). Additionally, the gut microbiota significantly influences these processes. Specific bacterial species such as *Bacteroides fragilis* and Erysipelotrichaceae are associated with enhanced immunogenicity and improved chemotherapy outcomes, while others like *Fusobacterium nucleatum* may contribute to immune suppression and poorer prognosis.^58^ These interactions highlight the intricate crosstalk between chemotherapy, the epithelial barrier, and the immune system, where both microbial composition and immune cell dynamics play crucial roles in determining therapeutic efficacy and patient outcomes. Understanding the interplay between the gut microbiome, immune response, and TME is pivotal in developing more effective therapeutic strategies for PDAC and other cancers.

### 2.4. The microbiome’s role in mediating chemotherapy toxicity and side effects

Chemotherapy, a long-standing cornerstone of cancer treatment, often leads to side effects such as diarrhea, mucositis, anemia, easy bruising, and hair loss, significantly impacting patients’ quality of life.^139,140^ Although not studied extensively, an increasing number of studies suggest a gut microbiome’s role in modulating the severity of these chemotherapy-induced toxicities.

The gut microbiome influences drug metabolism, and changes in its composition may alter the toxicity profile of drugs. As reviewed by Lo et al. (2023), the microbiome, particularly butyrate-producing bacteria, has been shown to mitigate 5-FUinduced toxicity by suppressing mucositis and diarrhea.^141^ Species like *Akkermansia muciniphila* and *Lactiplantibacillus plantarum* have been identified for their ability to suppress 5-FU-induced side effects through mechanisms such as restoring intestinal barrier function and suppressing eosinophil peroxidase and MPO activities.^142,143^ Furthermore, bacteria producing gmGUS can increase irinotecan toxicity, particularly diarrhea, by reactivating the prodrug SN-38.^56,57^ Chemotherapy drugs also can damage the mucosal lining of the gastrointestinal tract, causing mucositis.^144,145^ A healthy microbiome contributes to gut barrier integrity and can offer protection against mucosal damage.^146^ Lastly, the altered activity and expression of drug-metabolizing enzymes, which depend on microbial expression, are key in chemotherapy-induced toxicity.^147^ The composition and activity of the microbiome are thus crucial factors in managing the side effects of chemotherapy.

To date, there have been no additional studies specifically identifying the role of the microbiome in toxicities induced by gemcitabine or FOLFIRINOX in PDAC patients. This gap in research highlights a significant area for future investigation. The existing evidence that the microbiome can either suppress or exacerbate chemotherapy induced toxicity underscores the need for more studies to understand its specific role in the context of PDAC treatment. Understanding the microbiome’s interaction with chemotherapeutic agents, especially in PDAC, could be crucial for developing strategies to mitigate adverse effects. By leveraging this knowledge, medical professionals could aim to restore or preserve a healthy microbiome, thereby potentially reducing treatment-related toxicities. This approach could lead to improved patient outcomes, not only in terms of cancer management but also in enhancing overall quality of life during treatment.

## 3. Translational approaches: from laboratory research to clinical application

The advancement of precision medicine underscores the importance of the microbiome as a critical factor and a new potential target to enhance the efficacy and safety of patient-specific treatments. This approach is particularly relevant due to the notable inter-individual variability observed in responses to pharmacological treatments.^148^ The role of the gut microbiome in drug metabolism, initially recognized in Peppercorn and Goldman’s work in 1972,^149^ has been further illuminated with the emergence of ’pharmacomicrobiomics’. Coined by Saad et al. (2012), this term describes the complex relationship between the gut microbiome and drug reactions.^33^ This relationship significantly affects pharmacokinetics – altering drug absorption, distribution, metabolism, or elimination – and pharmacodynamics, which entails modifications in drug targets or biological pathways influencing sensitivity to pharmacological effects.^150,151^ One key aspect is the potential use of microbes and metabolites as predictive biomarkers. This approach could provide valuable information about how individuals might respond to certain drugs, paving the way for more tailored and effective treatments. Additionally, exploring strategies to regulate the gut microbiome presents a promising avenue for therapeutic interventions. By manipulating the microbiome, it might be possible to mitigate adverse drug reactions, enhance the effectiveness of treatments, and improve overall patient outcomes.

### 3.1. Microbes and metabolites as predictive biomarkers

The expanding area of microbiome research in PDAC underscores the pivotal role of the microbiome in shaping patient prognosis and treatment responses. Despite advances in understanding the microbiome’s influence in other cancers, PDAC lacks reliable biomarkers for predicting drug efficacy, often due to late-stage diagnosis and advanced tumor progression.^14^ Therefore, there is a critical need for research that identifies changes in the intestinal microbiome and correlates them with clinical outcomes at various stages of PDAC development. Some preclinical studies have established the mechanistic foundation for comprehending the microbiome’s impact on cancer therapy, with human association studies reinforcing these findings by correlating specific microbial patterns with treatment responses, prognosis, and the incidence of adverse effects.^151^ Nevertheless, further studies are essential to understand these associations and their potential implications for early detection and treatment strategies.

The study by Mendez et al. (2019) investigates the potential of the gut microbiome and its metabolic products as early detection tools for PDAC.^152^ Using a genetically engineered PDAC murine model, the study analyzes gut fecal microbiome through 16S rRNA pyrosequencing and whole-genome sequencing. The results revealed a dominance of Proteobacteria and Firmicutes in gut microbiome during the early stages of PDAC. Metabolic reconstruction showed an increase in polyamine and nucleotide biosynthetic pathways, which are assimilated by the host and utilized by rapidly dividing cells, signifying their role in tumorigenesis. Elevated serum polyamine levels in both mice and PDAC patients were observed, suggesting a strong correlation between microbial changes and metabolites fostering tumorigenesis. This study proposes the potential of microbial alterations and metabolites as noninvasive tools for early PDAC detection, which could improve patient outcomes.

The study by Nagata et al. (2022) focuses on identifying microbial signatures in the gut and oral microbiomes that can predict PDAC.^153^ Conducting a multinational study, they used shotgun metagenomic sequencing of fecal and salivary samples from treatment-naïve PDAC patients and non-PDAC controls across Japan, Spain, and Germany. The research uncovered significant dysbiosis in both gut and oral microbiomes and identified specific microbial species associated with PDAC. The study successfully constructed and validated metagenomic classifiers to predict PDAC, achieving high predictive accuracy across different cohorts. It also identified bacteriophages that infect PDAC-associated microbial species. This research presents the potential of metagenomics in providing robust biomarkers for PDAC identification and prognosis, indicating a global microbial signature for PDAC across diverse populations.^153^

**Figure 2.**
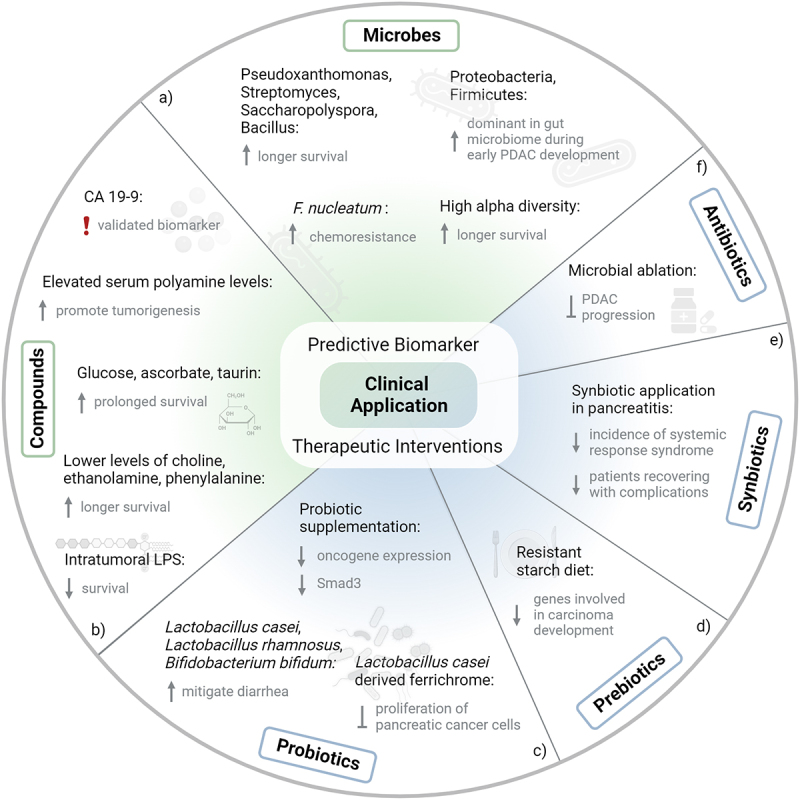
Clinical applications of microbiome studies (a) the composition of the gut and tumor microbiome can give indications for individual disease and therapy outcome. (b) Elevated or decreased concentrations of metabolic compounds can be associated with tumorigenesis and survival. CA 19–9 is an already validated biomarker for PDAC detection. (c) Using probiotics as therapeutic intervention, side effects like diarrhea can be mitigated. Also, proliferation of pancreatic cancer cells can be inhibited. (d) Resistant starch diet serves as prebiotic, leading to downregulation of genes involved in carcinoma development. (e) Synbiotics can reduce the incidence of system response syndrome and reduce complications during recovery in pancreatitis. (f) Altering the composition of the microbiome by specific antibiotics can inhibit PDAC progression (in mouse models).

Mitsuhashi et al. (2015) uncovered the prognostic significance of the tumor microbiome in PDAC, finding that the presence of *Fusobacterium* species in about 10% of cases correlated with a significantly worse prognosis.^90^ Riquelme et al. (2019) analyzed the tumor microbiome in PDAC patients with varying survival rates, discovering that long-term survivors exhibited higher alpha-diversity in their tumor microbiome.^99^ They identified a distinct microbiome signature, including *Pseudoxanthomonas*, *Streptomyces*, *Saccharopolyspora*, and *Bacillus clausii*, predictive of longer survival. This suggests the tumor microbiome’s diversity and composition can influence immune infiltration and PDAC survival, irrespective of the various therapies that have been studied. Mendez et al. (2020) reported a dominance of Proteobacteria and Firmicutes in the gut microbiome during early PDAC development^152^ (Figure 2a). Their study identified an increase in polyamine and nucleotide biosynthetic pathways in the altered microbial community. These metabolic products, assimilated by the host and utilized by proliferating cells, highlight their role in tumorigenesis. Elevated serum polyamine levels were observed in KPC mice and PDAC patients, linking microbial changes to metabolites that promote tumorigenesis. These findings suggest the potential for using microbial alterations as a noninvasive early detection method for PDAC, which could improve patient outcomes.

Features within the TME that can be impacted by specific bacterial compounds are also linked to clinical outcomes (Figure 2b). Guenther et al. (2020) conducted a study on advanced PDAC patients undergoing chemotherapy, including gemcitabine.^154^ They investigated the presence of lipopolysaccharide (LPS), linked to gram-negative bacteria, in tumor tissues. The study, involving 130 patients and a validation cohort of 113, found LPS in 24% of the samples. Patients with LPS-positive tumors had a notably shorter median overall survival of 4.4 months versus 7.3 months in those with LPS-negative tumors. This suggests that intratumoral LPS, a sign of gram-negative bacterial colonization, negatively impacts the effectiveness of gemcitabine in advanced PDAC. In a related study, Mayerle et al. (2018) analyzed the metabolic profiles of blood samples from a cohort of 914 individuals, including PDAC patients, chronic pancreatitis (CP) patients, liver cirrhosis patients, healthy subjects, and non-pancreatic disease controls.^155^ They identified a biomarker signature of nine metabolites, alongside CA 19–9, that could reliably distinguish PDAC from CP, with a remarkable 99.9% negative predictive value for cancer. Controversially, another study by Battini et al. (2017) involved 106 PDAC patients who underwent surgery.^156^ Metabolomics analysis of tissue specimens revealed that higher levels of glucose, ascorbate, and taurine were positively correlated with prolonged survival. Furthermore, those with longer survival tended to have lower levels of various metabolites including choline, ethanolamine, and phenylalanine, among others. This suggests a potential for using metabolic biomarkers to predict long-term survival in PDAC patients.

Understanding the role of microbes and metabolites is crucial in PDAC management. While CA 19–9 remains the primary validated biomarker in clinical routines for pancreatic cancer, new findings highlight the significant influence of the tumor microbiome on patient outcomes and treatment responses in PDAC. The identification of specific microbial signatures and metabolite pathways associated with PDAC development and progression provides critical insights for early detection and intervention, which could lead to better patient outcomes.

### 3.2. Regulating the gut microbiome: insights into therapeutic interventions

Currently, there is a spectrum of multifaceted approaches directed toward the modulation of the gut microbiome, encompassing a diverse array of strategies. These strategies include microbial manipulation via dietary alterations (such as probiotics, prebiotics, synbiotics) and antibiotics. In the context of PDAC, there are relatively few studies, but those that exist offer intriguing and potentially significant conclusions.

#### 3.2.1. Probiotics, prebiotics and synbiotics

The intricate relationship between the gut microbiome and cancer has garnered significant attention in recent years, particularly regarding the roles of prebiotics, probiotics, and synbiotics. These interventions hold promise for modulating the immune system and improving therapeutic outcomes in various malignancies, including PDAC. To provide a clear understanding of these concepts, Table 2 summarizes the definitions, examples, mechanisms of action, and potential benefits of prebiotics, probiotics, and synbiotics in the cancer context.

**Table 2.** Summary of prebiotics, probiotics, and synbiotics.

Concept	Definition	Examples	Mechanisms of Action	Health Benefits
Probiotics ^157–162^	Live microorganisms that confer health benefits when consumed in adequate amounts	*L. acidophilus, L. rhamnosus, L. casei L. reuteri, L. plantarum, B. infantis, B. longum B. lactis*, *S. boulardii, S. lactis*	Mutagen binding, degradation and mutagenesis inhibition, prevention of nontoxic procarcinogen conversion to harmful, toxic and highly reactive carcinogens, modulation and enhancement of the host’s innate immunity through the secretion of anti-inflammatory molecules	Modulate gut microbiota, enhance gut barrier function, produce antimicrobial substances, enhance immune response and prevent various gastrointestinal issues such as diarrhea
Prebiotics ^163–167^	Non-digestible food ingredients that promote the growth of beneficial bacteria	Inulin,Fructooligosaccharides (FOS),Galac- tooligosaccharides (GOS),Resistant Starch, Pectin	Stimulation of beneficial indigenous gut bacteria, production of SCFAs and lactic acid, as fermentation products, enhanced micronutrient absorption in the colon, modulation of xenobiotic metabolizing enzymes, modulation of immune response	Improve digestion, promote the growth and activity of beneficial gut bacteria, lead to increased production of immunoglobulins, improve overall immune function
Synbiotics ^168–172^	Combination of prebiotics and probiotics that synergistically improvegut health	Probiotics + Inulin (e.g., Lactobacillus + Inulin)	Combine the benefits of prebiotics and probiotics	Synergistic effect: enhanced gut health, improved digestion, and stronger immune function

Probiotics, defined as live microorganisms that provide health benefits when administered in adequate amounts, have shown promising results in immunomodulation in both preclinical models and cancer patients (Figure 2c). Chen et al. (2020) explored this in murine models, focusing on the interaction between probiotics and *Porphyromonas gingivalis*.^173^ They found that mice receiving both *P. gingivalis* and probiotics had significantly smaller pancreatic masses compared to those treated with *P. gingivalis* alone. This was accompanied by a decrease in oncogene expression and a reduction in both Smad3 and its phosphorylated form in the pancreatic tissue of these mice. Yeung et al. (2015) demonstrated that *Lactobacillus casei*, *Lactobacillus rhamnosus*, and *Bifidobacterium bifidum* can mitigate chemotherapy-induced diarrhea in murine models.^174^ They do this primarily by downregulating the expression of tumor necrosis factor alpha (TNF-*α*), interleukin-1*β* (IL-1*β*), and interleukin 6 (IL-6) mRNA. Furthermore, Kita et al. (2020) identified that ferrichrome, a compound derived from *Lactobacillus casei*, inhibits the proliferation of pancreatic cancer cells, including those resistant to 5-FU, as shown in both *in vitro* and *in vivo* mouse xenograft model experiments.^175^ These studies suggest that probiotics can influence cellular signaling pathways, particularly the mitogen-activated protein kinase (MAPK) and NF-*κ*B pathways, as noted by Thomas and Versalovic (2010) and Rahmani et al. (2023).^158,176^ The immunomodulatory effects and alterations in the gut microbiome composition offered by probiotics present beneficial outcomes, particularly in the context of cancer treatment and management.

Prebiotics, non-digestible food components that selectively stimulate the growth and/or activity of beneficial gut bacteria, have shown promise in cancer therapy.^163,177^ Trivieri et al. conducted a study using a xenograft mouse model and gene expression data (GSE16515) to investigate the effects of a prebiotic resistant starch diet (RSD) on miRNA expression profiles in pancreatic tumor tissues^178^ (Figure 2d). They discovered that a diet high in RSD led to the dysregulation of 19 miRNA genes compared to a control group. Further analysis using ingenuity pathways revealed that genes involved in carcinoma development, inflammatory response, abdominal cancer, metabolic disease, growth, invasion, and metastasis were downregulated in mice fed with RSD. Additionally, genes related to carbohydrate synthesis, glucose metabolism disorder, and cancer cell death were significantly upregulated. Another recent study on the impact of dietary prebiotics, specifically resistant starch (RS), on PDAC using xenograft mouse models demonstrated that a high RS diet led to the differential expression of genes associated with insulin receptor signaling, circadian rhythm signaling, and cancer drug resistance. Metabolomic analysis showed significant alterations in serum metabolites, including a reduction in purine compounds and an increase in glutamine levels. These changes suggest that RS influences carbohydrate and lipid metabolism, enhances fatty acid oxidation, and modulates tumor cell cycle and apoptosis-related genes, potentially working synergistically with existing cancer therapies. Furthermore, RS was found to regulate circadian rhythm genes, counteracting a typical deregulation observed in PDAC. Overall, the findings highlight the potential of resistant starch as an additive dietary intervention to modulate gene expression and metabolic profiles, thereby supporting conventional treatments and improving therapeutic outcomes in PDAC.^179^

The exploration of synbiotics (a combination of prebiotics and probiotics) in the treatment of cancer is an emerging area of interest, but currently, there is a notable gap in the literature specifically addressing their use in PDAC. However, insights can be drawn from studies on acute pancreatitis, which may provide some preliminary understanding of the potential benefits of synbiotics in pancreatic diseases (Figure 2e). A study in the context of acute pancreatitis is a prospective, randomized, double-blind study that compared outcomes in patients receiving only prebiotics (including inulin, beta-glucan, resistant starch, and pectin) against those receiving both prebiotics and a combination of four different *Lactobacillus* preparations with 10^10^ CFU. In the group receiving synbiotics, significant improvements were observed, including a lower total incidence of systemic response syndrome, reduced rate of late (over 48 hours) organ failure, and fewer patients recovering with complications. Additionally, there were nonsignificant trends toward a lower incidence of multiorgan failure, septic complications, and mortality.^180^ While chronic pancreatitis is known to be associated with the development and progression of pancreatic cancer, extrapolating findings from acute pancreatitis to PDAC requires caution due to potential biases.^181^ Notably, Maher et al. (2024) evaluated the immunomodulatory effects of synbiotics, compared to probiotics alone, in patients undergoing pancreaticoduodenectomy for PDAC. Ninety patients were randomly assigned to receive either a placebo, probiotics, or synbiotics (a combination of probiotics and inulin prebiotics) for two weeks preoperatively and one month postoperatively. The results demonstrated that the synbiotics group showed a significant increase in CD8+ T cell infiltration and IFN-*γ* expression in tumor tissues compared to the probiotics and placebo groups. Additionally, inflammatory cytokine levels (IL-1*β*, IL-6, and IL-10) were significantly reduced in the synbiotics group, indicating a pronounced anti-inflammatory effect. Furthermore, the synbiotics group experienced fewer postoperative complications, including reduced rates of anastomotic leakage, diarrhea, and abdominal distension, as well as a notable decrease in bacteremia.^182^ These findings suggest that synbiotics can enhance immune responses and improve postoperative outcomes in PDAC patients, highlighting their potential as an adjunctive therapy in the management of this malignancy.

Despite the increasing understanding of the microbiome’s role in cancer and the potential benefits of probiotics, prebiotics and synbiotics in other conditions, the application in PDAC remains largely unexplored.

#### 3.2.2. Antibiotics

Systemic antibiotics have demonstrated promising antitumoral effects in various preclinical models, suggesting a potential link between microbiome modulation and cancer progression. Key studies in this domain include those by Thomas et al. (2018), Sethi et al. (2018) and Pushalkar et al. (2020), which explore the relationship between the microbiome and tumor development in different cancer types, including PDAC.^126,183,184^ For example, Pushalkar et al. discovered that the ablation of the microbiota using an antibiotic cocktail consisting of vancomycin, neomycin, metronidazole, and amphotericin significantly enhanced the effectiveness of immune checkpoint blockade (ICB) in the treatment of PDAC in a mouse model. The study reported that the removal of the microbiome suppressed the development of both pre-invasive and invasive PDAC. However, transferring bacteria from tumor-bearing hosts promoted tumor growth. The elimination of bacteria was linked to an immunogenic reprogramming of the TME in PDAC. This included a decrease in myeloid-derived suppressor cells (MDSCs) and an increase in M1 macrophages, leading to the Th1 differentiation of CD4+ T cells and activation of CD8+ T cells. These findings suggest that endogenous microbes contribute to the immunosuppressive TME in PDAC, and that microbial ablation could be a promising strategy to inhibit PDAC progression^126^ (Figure 2f). Although numerous studies have demonstrated the effects of antibiotics in microbiome modulation using immunotherapies, the impact of these interventions on human pancreatic conditions, particularly in the context of chemotherapy and PDAC, remains unclear, under explored, and the results are still controversial.

Weniger et al. (2021) investigated the impact of the microbiome, particularly the gammaproteobacteria *Klebsiella pneumoniae*, on the progression of PDAC and the potential for quinolone treatment to mitigate its effects.^185^ Analyzing a cohort of 211 PDAC patients, the research revealed that an increase in the number of pathogen species in intraoperative bile cultures correlated with reduced progression-free survival (PFS). Gemcitabine improved PFS in patients without *K. pneumoniae* but not in those with positive cases. Notably, quinolone treatment was associated with enhanced overall survival (OS), regardless of *K. pneumoniae* status, and particularly benefited *K. pneumoniae*-positive patients. Patients with quinolone-resistant *K. pneumoniae* had shorter PFS. In conclusion, this study highlights the potential role of specific microbiome, especially *K. pneumoniae*, in chemoresistance and underscores the promise of targeted antibiotic treatment in PDAC management.

Fulop et al. (2023) aimed to investigate the impact of perichemotherapy antibiotics on the survival of patients with metastatic PDAC receiving first-line gemcitabine or fluorouracil chemotherapy.^186^ Analyzing data from 3,850 PDAC patients between 2007 and 2017, the study found that 56.6% of patients received antibiotics. Within this subgroup of 1,741 patients, 93.3% received antibiotics with gram-negative coverage, which was not associated with differential survival outcomes compared to patients who received antibiotics without gram-negative coverage (HR, 1.00; 95% CI, 0.83–1.21; *p >* 0.99). The majority of antibiotics administered were non-penicillin *β*lactams (50.5%) or fluoroquinolones (42.2%), with a higher proportion of fluorouracil treated patients receiving non-penicillin *β*-lactams (71.2%). Non-penicillin *β*-lactams were associated with an 11% reduction in the risk of death (HR, 0.89; 95% CI, 0.810.97; *p* = 0.01) compared to all other antibiotic classes, whereas fluoroquinolones alone were not associated with a difference in overall survival (HR, 1.00; 95% CI, 0.93–1.12; *p* = 0.70). Propensity-matched analysis revealed that antibiotic use was also associated with a 16% improvement in cancer-specific survival among gemcitabine-treated patients. However, no significant survival benefit was observed for antibiotic use in fluorouracil-treated patients. These findings underscore the potential role of antibiotics in modulating bacteria-mediated gemcitabine resistance, suggesting that strategic antibiotic use could enhance the efficacy of chemotherapy and improve patient outcomes in metastatic PDAC.

Another retrospective study of PDAC patients revealed that over 60% of those who underwent resection or had metastatic disease used antibiotics. The frequency of antibiotic use from diagnosis to death or the date of the last follow-up was 82% (*n* = 195), while forty-three patients (18%) did not receive any antibiotics. The following antibiotics were received by the resected PDAC patients: quinolones (*n* = 168), *β*lactams (*n* = 80), nitroimidazoles (*n* = 48), glycopeptides (*n* = 32), tetracyclines (*n* = 18), macrolides (*n* = 14), and sulfa drugs (*n* = 4).^187^ This group, when compared to those not exposed to antibiotics, showed increased OS of 13.3 months versus 9.0 months, and PFS of 4.4 months compared to 2 months in metastatic cases. Additionally, antibiotic use has been linked to enhanced effectiveness of gemcitabine-based treatments for PDAC.^188,189^

These findings collectively suggest that strategic antibiotic use could be a promising adjunct to enhance chemotherapy efficacy and improve outcomes in metastatic PDAC. However, the impact of these interventions in human pancreatic conditions remains under explored and controversial, necessitating further research to clarify these relationships.

## 4. Advancing microbiome research in pancreatic ductal adenocarcinoma: overcoming current challenges

The horizon of cancer therapies has witnessed the emergence of novel insights and investigations poised to serve as alternative approaches. Among these promising avenues are fecal microbiota transplantation (FMT), the development of engineered microbial consortia, and personalized phage therapy.^8,12,190,191^ These innovations shed light upon their evolving roles in the reconfiguration of the gut microbiome landscape and their potential ramifications within the sphere of cancer treatment. However, many of these alternatives have not yet been tested for PDAC, and therefore the area still lacks more research and innovation. As we delve into the evolving landscape of PDAC research and treatment, several pioneering approaches are emerging that have the potential to reshape our understanding and management of this challenging disease. These include the development and application of machine learning models for early detection, the intricate analysis of low-biomass microbiomes in cancer tissues, and the exploration of microbe-host interactions through advanced model systems. We will explore these topics in detail in the upcoming sections.

### 4.1. Machine learning tools for early cancer detection and treatment prediction

In recent years, artificial intelligence and particularly machine learning algorithms are becoming increasingly important in the field of biology and microbiome studies, particularly in complex disorders.^192^ These technologies enable detailed analysis of very large datasets from microbiome research, revealing insights in an unsupervised and supervised manners. Machine learning methods are crucial for integrating largescale microbiome data with clinical and molecular features, in order to predict tumor development, progression, and response to treatment. Thereby, these methods can aid in implementing personalized treatment strategies.

Several recent publications show the successful usage of machine learning algorithms for predicting PDAC prognosis. For example, the work by Jia et al. (2023) focused on developing a pancreatic cancer risk prediction model (Prism) using Electronic Health Record (EHR) data from a multi-institutional federated network.^193^ This model aimed at early detection of PDAC 6–18 months before diagnosis. The prediction model, including deep neural network-based model coined PrismNN and a variation of linear model PrismLR, was developed using data from 55 US Health Care Organizations, targeting patients aged 40 years or older. The study found Prism models to have good accuracy and generalizability across different populations, suggesting their potential utility in early PDAC detection, thus expanding the scope of current screening guidelines.^193^ In another approach, Placido et al. (2023) focuses on developing a deep learning algorithm to predict the risk of PDAC from disease trajectories.^194^ The study utilized clinical data from millions of patients in Denmark and the US to train machine learning models on sequences of disease codes in clinical histories. These models were used to predict cancer occurrence within various time windows. The best-performing model demonstrated high predictive accuracy, highlighting the potential of this technology in early cancer detection and surveillance program design.^194^ Another work by Cao et al. (2023) presented a deep learning model named PANDA (Pancreatic Cancer Detection with Artificial Intelligence) that significantly improves the detection and diagnosis of PDAC and other pancreatic lesions through non-contrast CT scans.^195^ PANDA was trained on a large dataset of patients from a single center and validated across multiple centers. It showed remarkable accuracy, outperforming radiologists in detecting lesions and identifying PDAC, with high sensitivity and specificity.^195^

It has been also shown that estimations from the gut microbiome data for PDAC, Kartal et al. (2022) investigated the potential of fecal microbiomes as a non-invasive diagnostic biomarker for PDAC.^196^ Utilizing shotgun metagenomic and 16S rRNA amplicon sequencing, fecal and salivary microbiome from PDAC patients, chronic pancreatitis patients, and controls were studied, and fecal metagenomic classifiers, especially those based on 27 microbial species, could accurately identify PDAC with high specificity. Combining these classifiers with the CA19–9 serum marker, the only FDA-approved PDAC diagnostic biomarker, significantly improved diagnostic accuracy. This research highlights the feasibility of using fecal microbiota-based screening for early detection of PDAC.^196^

While machine learning offers promising tools for the integrated analysis of various data types, researchers face several challenges when working with microbiome data:

- **Data Availability**. The high cost of DNA analysis, particularly for metagenomics, and the sporadic nature of disease occurrences often result in microbiome datasets with lower sample sizes.^197,198^ To address this issue, strategies like utilizing pretrained models or generating synthetic data can help provide more examples.
- **Model Performance**. Accurate model development is crucial. For microbiome data, decision-tree-based approaches such as random forest^199^ and gradient boosting^200^ are preferred. They manage complex relationships and enhance performance through ensemble techniques. Rigorous testing, including cross validation, is vital for ensuring model reliability.
- **Explainability**. Understanding model predictions in microbiome analysis is crucial.^201^ Techniques like SHAP (SHapley Additive exPlanations)^202^ and LIME (Local Interpretable Model-agnostic Explanations)^203^ offer visual insights, thereby increasing transparency by highlighting how specific features impact predictions.
- **Reproducibility**. Ensuring reproducibility is challenging due to variations in data sharing practices. Comprehensive data preprocessing, robust data pipelines, and strict version control for data and models are essential. Tools like Docker^204^ facilitate managing software dependencies and documenting all workflow steps enhances reproducibility.

Despite these challenges, leveraging machine learning in microbiome research holds transformative potential for early cancer detection and treatment prediction, particularly in diseases like PDAC. As methodologies improve and integration deepens, these tools are increasingly important for developing personalized treatment strategies that are informed by detailed, data-driven insights into individual microbiome profiles.

### 4.2. Low-Biomass microbiome analysis in cancer tissues

The advent of next-generation sequencing (NGS) has revolutionized our ability to analyze microbiomes, including those present in human cancer tissues and formalin-fixed paraffin-embedded (FFPE) tissues. These low-biomass microbiome samples provide valuable insights into the microbial environment of tumors. However, the analysis of such samples presents specific challenges due to the inherently low concentration of microbial DNA.

A significant issue in low-biomass microbiome analyses is contamination, which can overshadow the actual microbial signal from the samples. Bacterial contaminants introduced during sample collection, DNA extraction, and library preparation can generate substantial background noise, making it difficult to discern the true microbial profile within tissues.^205^ This issue is exacerbated in medical samples, where the lack of appropriate controls further complicates the analysis.^206^ To address these challenges and enhance the accuracy of low-biomass microbiome analyses, methodological approaches have been proposed and implemented. These strategies aim to minimize contamination and improve the detection of the actual microbial DNA present in the samples. Such approaches might include rigorous control of the laboratory environment, the use of reagents and materials with minimal microbial DNA contamination, and the implementation of specific bioinformatic tools to distinguish between contaminant and sample-derived sequences.

The RIDE (Report, Include, Determine, Explore) criteria, proposed by Eisenhoffer et al. (2019), represent a comprehensive approach to mitigate bacterial contamination in low-biomass microbiome studies.^207^ These criteria have become particularly important in ensuring the accuracy and reliability of microbiome studies, especially those involving low-biomass samples. In addition to the RIDE criteria, Dohlman et al. (2021) suggested including negative and positive controls in studies to differentiate between bacterial contaminants and the biological samples of interest.^208^ These controls are crucial in discerning true biological signals from contamination artifacts.

To aid in filtering bacterial contaminants from biological samples, bioinformatics tools such as R Decontam^209^ and SourceTracker^210^ have been developed. These programs are publicly available and provide researchers with sophisticated methods to identify and remove contaminant sequences. For instance, Poore et al. (2020) utilized DNA and RNA concentrations to identify potential bacterial contamination at the genus level. They then applied this information to remove these genera from negative blank reagents used as negative controls.^211^ Gihawi et al. (2023) emphasized the importance of including genera only when there is strong computational and biological evidence for their presence in the sample of interest.^212^ This careful consideration is essential given the high likelihood of bacterial contamination in low-biomass samples. However, they also highlighted that these computational methods for filtering contaminants are not substitutes for strict microbiological practices. Sterile processing in sample collection, DNA extraction, and library preparation for sequencing remains vital to prevent contamination and ensure the integrity of microbiome data.

Recent advancements in microbiome research have significantly enhanced our understanding of the microbial diversity and composition within solid tumors. Two notable studies exemplify the application of sophisticated techniques and controls to reliably profile intra-tumoral microbiomes, highlighting the diversity across different cancer types and refining the identification and exclusion of potential contaminants. Nejman et al. (2020) conducted a comprehensive study on 1,526 intra-tumoral microbiomes across seven solid tumor types.^213^ Using a 16S rRNA multi-region approach coupled with stringent negative controls, they implemented several filtering steps to address bacterial contamination. Their analysis revealed substantial differences in diversity and composition within these tumor types. This study is significant because it underscores the complex and varied nature of the tumor microbiome, which could have implications for understanding tumor biology, treatment responses, and potential therapeutic targets. Roelands et al. (2023) took a different approach to ensure the accuracy of microbiome analyses.^214^ They compiled and flagged likely bacterial contaminants, drawing on data from negative blank reagents described by Salter et al. (2014) and Poore et al. (2020).^205,211^ This list of potential contaminants was then used to eliminate these bacteria from the abundance matrix, thus refining the analysis of the true microbiome present in the samples. By excluding known contaminants, this method enhances the reliability of microbiome data, leading to more precise insights into the microbial ecology of tumors.

These studies represent significant strides in microbiome research within oncology, demonstrating the importance of rigorous methodologies and controls in studying low biomass environments such as tumors. They highlight the diversity of the tumor microbiome across different cancers and the necessity of careful consideration in identifying and excluding bacterial contaminants. By adhering to these practices, researchers can generate more accurate and reliable data, contributing to the growing understanding of the microbiome’s role in health and disease.

### 4.3. Experimental models to explore microbiome-host interactions in PDAC

Recent advancements in PDAC research have increasingly highlighted the importance of organoid technology and microbiome interactions in understanding tumor biology and treatment resistance. Organoids, particularly patient-derived organoids (PDOs), have revolutionized cancer research by providing three-dimensional cultures that closely mimic the *in vivo* tumor environment. These models have been instrumental in studying cancer pathogenesis, drug resistance, and treatment efficacy, offering a promising platform for personalized medicine. Studies in gastric cancer and CRC have led the forefront in organoid research, as indicated by the works of.^215–224^ However, integrating microbiome research with PDOs in PDAC presents unique challenges and opportunities. The tumor microbiome is now recognized as a significant factor in PDAC oncogenesis, influencing tumor growth, immune modulation, and chemotherapy resistance. Studies have shown that co-culturing PDOs with some bacteria can better replicate the TME, providing deeper insights into the complex interactions between tumor cells and the microbiome.^225–227^

A pivotal study by Chen et al. (2022) demonstrated the role of type I collagen (Col1) homotrimers in PDAC. The researchers found that pancreatic cancer cells produce a unique Col1 homotrimer (*α*1/*α*1/*α*1), which is absent in normal cells. This aberrant collagen variant promotes tumor growth and resistance to chemotherapy by engaging with the *α*3*β*1 integrin on cancer cells, subsequently influencing the tumor microbiome and immune landscape. Using PDOs, the study revealed that the presence of Col1 homotrimers enhances tumor cell proliferation and resistance to chemotherapeutic agents like gemcitabine. Additionally, gnotobiotic mouse models demonstrated that deletion of Col1 homotrimers resulted in a reprogrammed tumor microbiome, characterized by reduced Bacteroidales and increased Campylobacterales, which is associated with improved immune infiltration and response to anti-PD-1 immunotherapy.^228^ These findings underscore the importance of the tumor microenvironment, including the microbiome, in modulating cancer progression and treatment efficacy. By leveraging PDOs and gnotobiotic models, researchers can further dissect the molecular and microbial interactions that drive PDAC, offering potential avenues for targeted therapies that incorporate microbiome modulation.

Despite several advancements, significant challenges remain. One primary issue is the lack of standardized methods for co-culturing PDOs with bacteria, which can affect the reproducibility and reliability of results. The transcriptional landscape of PDOs can be heavily influenced by culture conditions, potentially altering their response to pharmacological interventions. Additionally, the complexity of the TME in PDAC, characterized by a dense stroma and low cellularity, makes it difficult to fully recapitulate it *in vitro*.

The use of gnotobiotic mouse models also has significantly contributed to our understanding of the microbiome’s role in oncogenesis and cancer therapy. These models, characterized by their germ-free status or a defined microbiota, provide a controlled environment for studying the impact of microorganisms on various aspects of cancer, including development, progression, and response to treatment. Gnotobiotic mice allow researchers to introduce specific bacteria or bacterial consortia and observe their direct effects on tumor growth, immune modulation, and treatment efficacy. This approach has been utilized in several studies, including those by.^190,229–232^ These studies have shed light on how particular microbial communities or metabolites can influence cancer dynamics. Despite their utility, the application of gnotobiotic mouse models in PDAC research is still relatively nascent. Nonetheless, these models hold great promise for advancing our understanding of PDAC, particularly in the context of microbial metabolites. For instance, Tintelnot et al. (2023) explored the impact of these metabolites on PDAC pathogenesis, offering valuable insights into the complex relationship between the microbiome and cancer.^101^ By understanding the specific interactions between the microbiome and PDAC, researchers can develop more personalized and effective cancer care strategies.

Finally, phage display technology, which involves using bacteriophages to discover peptides and antibodies with specific binding affinities, has been extensively utilized in cancer research and the exploration of bacteriophages as a therapeutic strategy for targeting intratumoral bacteria presents an innovative approach in cancer treatment, particularly in the context of PDAC. Bacteriophages, or phages, are viruses that selectively infect and destroy specific bacterial species. Their ability to precisely target certain bacterial populations makes them a promising tool for modulating the microbiome in a cancer-specific context. Recent research, including studies by Zheng et al. (2020), Li et al. (2023), and Dong et al. (2023), is investigating the potential of bacteriophages in both preclinical and clinical settings.^2,233,234^ The use of bacteriophages offers a targeted method to alter the gut microbiome, with therapeutic effects that can be confined to specific organs or tumor sites. PDAC poses unique challenges for effective drug delivery due to the tumor’s dense stroma and complex microenvironment. However, initial studies, such as those by Asar et al. (2020) and Kabwe et al. (2022), suggest that bacteriophages can successfully infiltrate pancreatic tumors.^235,236^ This capability could significantly enhance the delivery and efficacy of therapeutic agents in PDAC. Some products derived from this technology have even received FDA approval for cancer therapy, as highlighted by Passariello et al. (2020).^237^ Despite its success in other cancer types, the application of phage display and bacteriophage therapy in PDAC is still in its early stages.

## 5. Conclusions

The gut microbiome’s influence on chemotherapy efficacy, particularly in PDAC, hinges on several mechanisms, notably drug metabolism and immune modulation. Gut bacteria produce enzymes, which can activate or deactivate chemotherapy drugs, increasing efficacy, but also toxicity. Additionally, gut microbes can alter the bioavailability of chemotherapeutic drugs by modifying their absorption and distribution, potentially enhancing or diminishing drug efficacy. The gut microbiome also modulates the immune system, crucial for enhancing the effectiveness of chemotherapy. Certain gut bacteria can induce cytokine production, or T cell differentiation bolstering antitumor immune responses and thereby improving chemotherapy efficacy. Moreover, gut microbes preserve gut barrier integrity, regulate a host’s immune milieu and affects the tumor microenvironment which all impacts on chemotherapy tolerability, toxicity and efficacy. In the exploration of the microbiome’s influence on chemotherapy efficacy in PDAC, we’ve seen how advances in microbiome-based therapies, driven by synthetic biology and enhanced understanding of microbe-host interactions, are revolutionizing PDAC treatments.

Despite these promising developments, some findings remain inconclusive due to differences in study design, patient populations, and microbiome analysis methods. Although research in mouse models has provided insights into the microbiome’s effects on tumor development and immune system interactions, the variability in microbiome composition among individuals and species differences present challenges in translating these findings to humans. Also, the composition of the gut microbiome varies significantly between individuals, influenced by factors such as diet, genetics, and environment. This variability can lead to different interactions with chemotherapy drugs, making it challenging to generalize findings across diverse populations. These inconsistencies highlight the multifactorial nature of chemotherapy efficacy, influenced by genetics, tumor biology, and patient health status, making it difficult to isolate the impact of the gut microbiome. Another concern is the use of meticulous sampling, careful processing and precise analysis of microbial sequencing and metabolomic datasets. The potential of microbiome-targeted interventions in PDAC is promising, highlighting the need for further research to fully exploit the microbiome’s diagnostic and therapeutic capabilities. This evolving field offers new prospects for more personalized and effective chemotherapy strategies in PDAC treatment, representing a significant step forward in medical oncology.
